# Two-Trait Predictor of Venous Invasion on Contrast-Enhanced CT as a Preoperative Predictor of Outcomes for Early-Stage Hepatocellular Carcinoma After Hepatectomy

**DOI:** 10.3389/fonc.2021.688087

**Published:** 2021-09-01

**Authors:** Xinming Li, Xuchang Zhang, Zhipeng Li, Chuanmiao Xie, Shuping Qin, Meng Yan, Qiying Ke, Xuan Jin, Ting Lin, Muyao Zhou, Wen Liang, Zhendong Qi, Zhijun Geng, Xianyue Quan

**Affiliations:** ^1^Department of Radiology, Zhujiang Hospital, Southern Medical University, Guangzhou, China; ^2^Department of Radiology, Sun Yat-sen University Cancer Center, Guangzhou, China

**Keywords:** hepatocellular carcinoma, tomography, x-ray computed, two-trait predictor of venous invasion, hepatectomy, biomarkers, prognosis

## Abstract

**Objectives:**

This study aimed to assess the effectiveness of the two-trait predictor of venous invasion (TTPVI) on contrast-enhanced computed tomography (CECT) for the preoperative prediction of clinical outcomes in patients with early-stage hepatocellular carcinoma (HCC) after hepatectomy.

**Methods:**

This retrospective study included 280 patients with surgically resected HCC who underwent preoperative CECT between 2012 and 2013. CT imaging features of HCC were assessed, and univariate and multivariate Cox regression analyses were used to evaluate the CT features associated with disease-free survival (DFS) and overall survival (OS). Subgroup analyses were used to summarized the hazard ratios (HRs) between patients in whom TTPVI was present and those in whom TTPVI was absent using a forest plot.

**Results:**

Capsule appearance [HR, 0.504; 95% confidence interval (CI), 0.341–0.745; p < 0.001], TTPVI (HR, 1.842; 95% CI, 1.319–2.572; p < 0.001) and high level of alanine aminotransferase (HR, 1.620; 95% CI, 1.180–2.225, p = 0.003) were independent risk factors for DFS, and TTPVI (HR, 2.509; 95% CI, 1.518–4.147; p < 0.001), high level of alpha-fetoprotein (HR, 1.722; 95% CI, 1.067–2.788; p = 0.026), and gamma-glutamyl transpeptidase (HR, 1.787; 95% CI, 1.134–2.814; p = 0.026) were independent risk factors for OS. A forest plot revealed that the TTPVI present group had lower DFS and OS rates in most subgroups. Patients in whom TTPVI was present in stages I and II had a lower DFS and OS than those in whom TTPVI was absent. Moreover, there were significant differences in DFS (p < 0.001) and OS (p < 0.001) between patients classified as Barcelona Clinic Liver Cancer stage A in whom TTPVI was absent and in whom TTPVI was present.

**Conclusions:**

TTPVI may be used as a preoperative biomarker for predicting postoperative outcomes for patients with early-stage HCC.

## Introduction

Hepatocellular carcinoma (HCC) is the sixth most commonly diagnosed tumor and the fourth leading cause of cancer-related death worldwide (1). Hepatectomy and liver transplantation are the most effective treatments for HCC, and early-stage HCC has better prognosis after resection (2, 3). However, reports suggest that the 5-year recurrence rates after hepatectomy and liver transplantation are 75% and 25%, respectively (4). Therefore, an effective indicator for predicting and monitoring HCC in order to select the best candidates for surgical resection is urgently needed.

In addition to staging, tumor differentiation and microvascular invasion (MVI) are well-known accepted independent predictors of HCC (5–7). However, these pathological markers of tumor behavior can only be evaluated after surgery. Therefore, preoperative markers of tumor aggressiveness would help with patient selection before surgery. Serum alpha-fetoprotein (AFP) has been proposed as a routine clinical parameter to aid in the diagnosis of HCC and for monitoring recurrence and prognostic factors (8, 9). Computed tomography (CT) and magnetic resonance imaging (MRI) are routine examination methods in clinical practice, and they play important roles in diagnosis, staging, follow-up, and efficacy evaluation of HCC. However, the usefulness of preoperative CT and MRI has been greatly undervalued (8–10).

A two-trait predictor of venous invasion (TTPVI) consisting of “internal arteries” and “hypodense halos” was proposed by Segal et al. (11). It has been reported that TTPVI correlates to a specific HCC molecular profile, derived from a microscopic venous invasion gene profile associated with cellular proliferation, angiogenesis, and MVI (12). In fact, several studies have confirmed that TTPVI is strongly associated with MVI and has a high diagnostic performance in predicting MVI (12–16). However, to the best of our knowledge, whether the presence of TTPVI on contrast-enhanced CT (CECT) can serve as a prognostic factor in HCC after hepatectomy treatment has not been fully investigated.

Therefore, in the present study, we attempted to evaluate the impact of TTPVI detected on preoperative CECT on disease-free survival (DFS) and overall survival (OS) in patients with early-stage HCC who underwent hepatectomy.

## Materials and Methods

This retrospective clinical study was approved by the institutional ethics review board of Zhujiang Hospital of Southern Medical University (2019-KY-021-01) and Sun Yat-sen University Cancer Center (B2021-214-01), and the requirement for written informed consent was waived.

### Patients

Our surgical and histological database was reviewed to identify patients who underwent hepatic resection for HCC and preoperative CECT between January 2012 and December 2013. The inclusion criteria were as follows: (a) patients who underwent a CT scan not earlier than 1 month before surgery; (b) patients who were classified as early-stage HCC (stage 0 or A) based on the Barcelona Clinic Liver Cancer (BCLC) classification (17); and (c) patients with pathological confirmation of primary HCC and reported MVI status. Patients with prior surgical or medical treatment were excluded. A total of 280 patients (280 single HCCs) were included (248 male and 32 female; mean age, 50.8 years; range, 18–82 years), as shown in **Supplementary Figure 1**.

We collected preoperative laboratory data on the following: levels of albumin, alkaline phosphatase, aspartate aminotransferase, alanine aminotransferase (ALT), total bilirubin, gamma-glutamyl transpeptidase (GGT), AFP, and carbohydrate antigen 19-9, prothrombin time, hepatitis B virus (HBV) deoxyribonucleic acid, and immunology of HBV and hepatitis C virus.

Our protocol requirements for CECT met the criteria recommended by the American Association for the Study of Liver Diseases guidelines. Precontrast and CECT images were acquired by three CT scanners with multidetectors at both hospitals. CECT images including hepatic arterial, portal venous, and delayed phase images were obtained 30, 60, and 180 s after the injection of contrast enhancement material (Ultravist Iopromide 370 mgI/ml, Bayer-Schering Pharma; or Lopamicro 370 mgI/ml, Brocca Pharma) at a rate of 2–3 ml/s with a dose of 1–1.5 ml/kg bodyweight. The scanning parameters of each CT scanner are shown in **Supplementary Table 1**. The mean time between CT imaging and surgery was 10.9 ± 12.6 days (range, 0–90 days).

### Histopathological Evaluation

We collected the following pathologic data: Edmondson–Steiner grade, histological type, cell type, capsule invasion, presence of MVI, and bile duct invasion. We defined MVI as the presence of tumor emboli in a vascular space lined by endothelial cells that was visible only by microscopy (18, 19). We also evaluated the patients’ cancer stage based on the American Joint Committee on Cancer (AJCC) and BCLC stages.

### Image Analysis

All CT images were retrospectively assessed by two radiologists with 4 (QZ) and 9 (GZ) years of experience in hepatic imaging. The radiologists were aware that the patients had HCC but were blinded to the clinical and other pathological findings. The two reviewers independently evaluated the following imaging features for each HCC: (a) tumor diameter, (b) intratumor necrosis, (c) internal arteries (11, 12), (d) hypoattenuating halos (11, 12, 20), (e) radiological capsule appearance, (f) margin (12), and (g) TTPVI, which was defined as the presence of internal arteries in the arterial phase and hypoattenuating halos in the portal venous or delayed phases (11, 12). Imaging examples are shown in **Supplementary Figures 2**–**6**.

### Follow-Up After Surgical Resection

The patients were followed up with AFP examinations and CECT or MRI at intervals of 3–6 months after surgery. The duration of DFS or time until death was recorded. The patients were censored on the date on which communication was lost or on May 15, 2019, whichever came first. OS was calculated from the date of liver resection to the last follow-up or until death. DFS was defined as the interval between surgery and recurrence, metastasis (confirmed by CECT or MRI), or death from any cause.

### Statistical Analysis

All statistical tests were performed using R statistical software version 3.6.1 (http://www.r-project.org/). Continuous variables are expressed as the mean and standard deviation and were compared using the two-tailed *t*-test or Mann-Whitney *U*-test. Categorical variables are shown as the number of cases and were analyzed using the chi-square test or Fisher’s exact test. The interobserver agreement between the two reviewers assessing the CT imaging features was evaluated. The cumulative DFS and OS rates were estimated using the Kaplan–Meier method, and the differences between curves were evaluated using the log-rank test using the “survminer” package and “survival” package. A Cox proportional hazards regression analysis was used to assess the imaging features and clinical findings associated with DFS and OS in HCC. Variables with a p < 0.1 in the univariable analysis were included in the multivariable analysis. The “forestplot” package was used to perform forest plots.

## Results

### Patient Characteristics

Demographic data and baseline clinical characteristics of the study patients are presented in **Table 1**. TTPVI was detected on preoperative CECT in 124 (44.3%) patients, 162 (57.9%) patients had a radiological necrosis appearance, 96 patients had a radiological capsule appearance, and 154 (77.9%) patients had a smooth tumor margin. The median follow-up time was 1,991 days [interquartile range (IQR), 809–2,260 days]. In total, 76 patients died during follow-up (median time, 742 days; IQR, 359–1,337 days), and 158 patients had a recurrence (median time, 322 days; IQR, 136–738 days).

**Table 1:** Demographic data and baseline clinical characteristics of the study patients.

Variable	Value
**Age (years)**	50.8 ± 11.1
**Gender, n (%)**	
**Female**	32 (11.4)
**Male**	248 (88.6)
**MVI, n (%)**	115 (41.1)
**Edmondson–Steiner, n (%)**	
**I–II**	178 (63.6)
**III–IV**	102 (36.4)
**Cirrhosis, n (%)**	168 (60.0)
**Etiology, n (%)**	
**HBV**	255 (91.1)
**HCV**	1 (0.4)
**HBV and HCV**	2 (0.7)
**Other disease origins**	22 (7.9)
**PLT (10^9^/L)**	182.2 ± 70.5
**ALB (g/L)**	41.3 ± 4.3
**ALP (U/L)**	95.8 ± 58.4
**ALT (U/L)**	61.7 ± 78.1
**AST (U/L)**	55.5 ± 66.8
**GGT (U/L)**	83.1 ± 116.2
**TB (μmol/L)**	15.2 ± 7.2
**PT (s)**	12.1 ± 1.0
**AFP (ng/ml)**	6,266.3 ± 19,939.1
**CA19-9 (kU/L)**	29.7 ± 55.9
**Diameter (cm)**	5.7 ± 3.1
**Necrosis, n (%)**	162 (57.9)
**Capsule appearance, n (%)**	96 (34.3)
**Margin, n (%)**	126 (45.0)
**TTPVI, n (%)**	124 (44.3)
**BCLC, n (%)**	
**Stage 0**	17 (6.1)
**Stage A**	263 (93.9)
**AJCC, n (%)**	
**Stage I**	157 (56.1)
**Stage II**	123 (43.9)
**Postoperative TACE**	59 (21.1)

AFP, alpha-fetoprotein; ALB, albumin; ALP, alkaline phosphatase; ALT, alanine aminotransferase; AST, aspartate aminotransferase; CA19-9, carbohydrate antigen 19-9; GGT, gamma-glutamyl transpeptidase; HBV, hepatitis B virus; HCV, hepatitis C virus; MVI, microvascular invasion; PLT, platelets; PT, prothrombin time; TACE, trans-catheter arterial chemoembolization; TB, total bilirubin; TTPVI, two-trait predictor of venous invasion.

### Interobserver Agreement of CT Imaging Features

The interobserver agreement of CT imaging features is shown in **Supplementary Table 2**, which was good or excellent (κ = 0.818 for necrosis, κ = 0.843 for internal arteries, κ = 0.748 for halo, κ = 0.808 for capsule, κ = 0.799 for margin, κ = 0.787 for TTPVI).

### Prognostic Factors of DFS and OS for HCC

**Figure 1:**
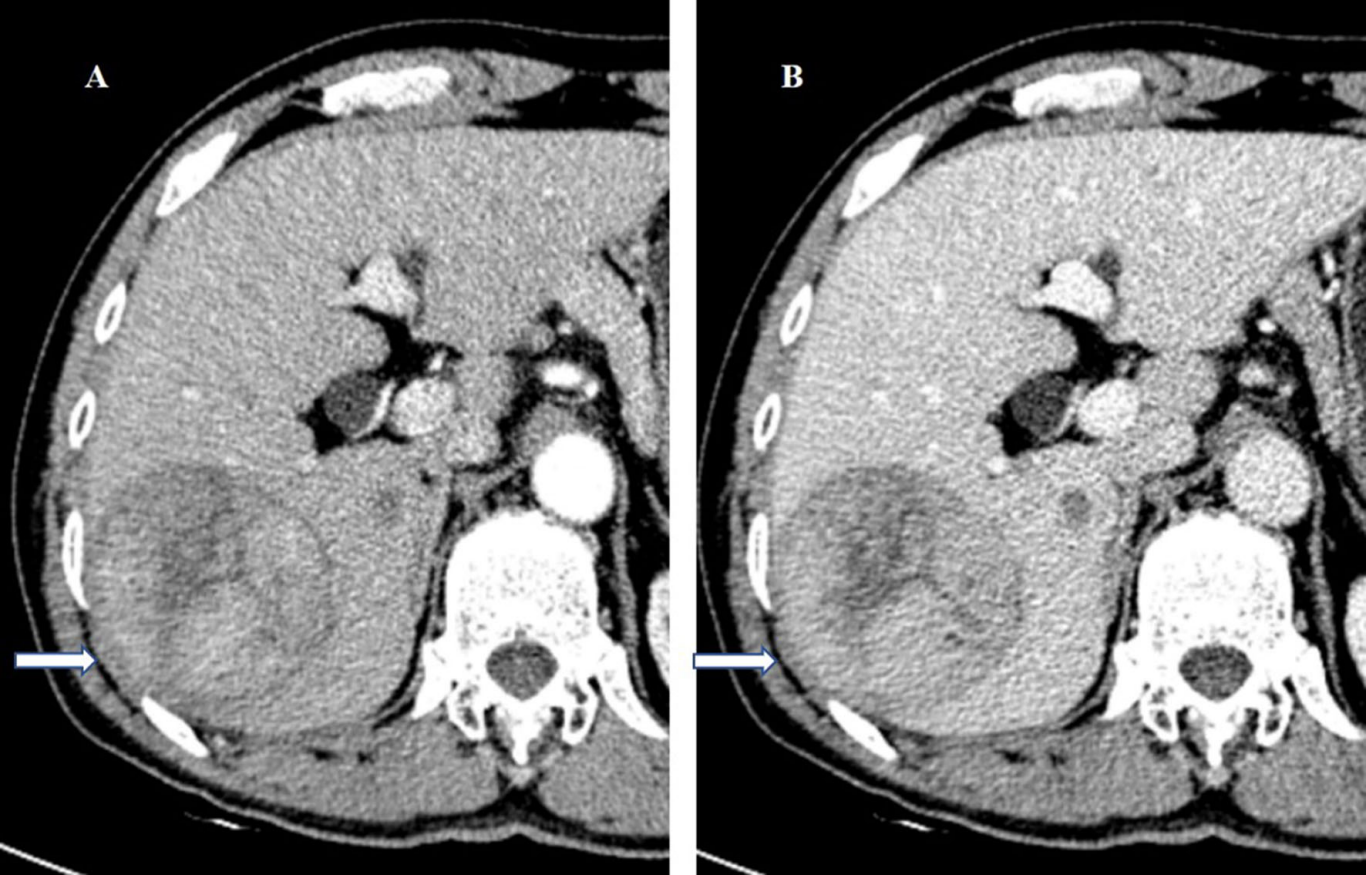
A 57-year-old man underwent surgery resection for HCC. **(A)** In the arterial phase image, the 71-mm liver mass shows hyperenhancement. **(B)** In the balanced phase image, the mass shows washout. HCC with TTPVI absent on the preoperative contrast-enhanced computed tomography was diagnosed. On histopathology after surgery resection, the tumor was classified as HCC without microvascular invasion. Tumor recurrence did not occur during the 75 months of follow-up after surgery resection. HCC, hepatocellular carcinoma; TTPVI, two-trait predictor of venous invasion.

**Figure 2:**
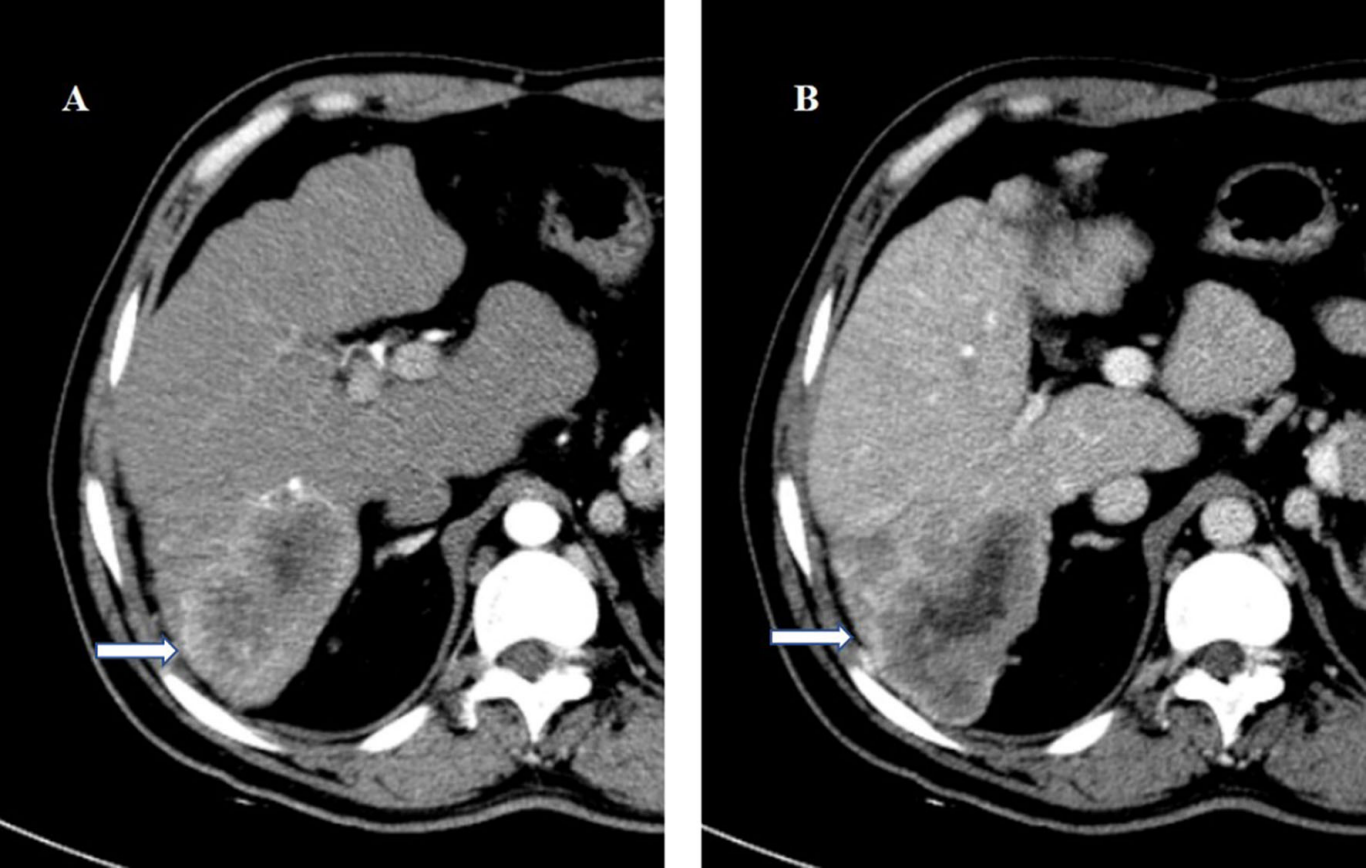
A 46-year-old man underwent surgery resection for HCC. **(A)** In the arterial phase image, the 72-mm liver mass shows hyperenhancement. **(B)** In the delayed phase image, the mass shows washout. HCC with TTPVI present on the preoperative contrast-enhanced computed tomography was diagnosed. On histopathology after surgery resection, the tumor was classified as HCC with microvascular invasion. Tumor recurrence occurred 6 months after surgery resection. The patient died 20 months after surgery resection. HCC, hepatocellular carcinoma; TTPVI, two-trait predictor of venous invasion.

**Figure 3:**
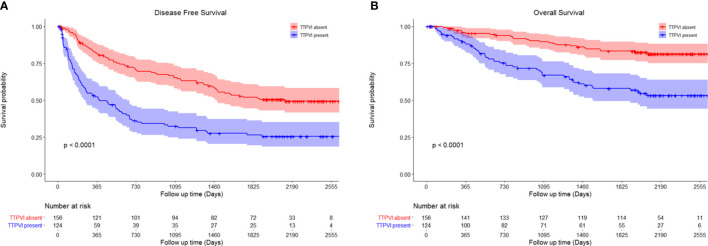
Kaplan–Meier curves of **(A)** disease-free survival and **(B)** overall survival between TTPVI present and absent groups. TTPVI, two-trait predictor of venous invasion.

All preoperative clinical and imaging features were included in the univariate and multivariate Cox regression analyses of DFS and OS. The hazard ratios (HRs) and 95% confidence intervals (CIs) and corresponding p-values of the univariate and multivariate analyses of DFS and OS are shown in **Tables 2**, **3**, respectively. On multivariate analysis, radiological capsule appearance (HR, 0.504; 95% CI, 0.341–0.745; p < 0.001), TTPVI (HR, 1.842; 95% CI, 1.319–2.572; p < 0.001), and high level of ALT (HR, 1.620; 95% CI, 1.180–2.225; p = 0.003) were independent risk factors for DFS, while TTPVI (HR, 2.509; 95% CI, 1.518–4.147; p < 0.001), high level of AFP (HR, 1.722; 95% CI, 1.067–2.788; p = 0.026), and GGT (HR, 1.787; 95% CI, 1.134–2.814; p = 0.026) were independent risk factors for OS. Recurrence of HCC was observed in 85 patients (68.6%) of the TTPVI present group (**Figure 1**) and 73 patients (46.79%) of the TTPVI absent group (**Figure 2**). The 1-, 2-, and 3-year DFS rates were 54.84%, 37.52%, and 33.67%, respectively, for patients in whom TTPVI was present and 81.08%, 69.99%, and 65.54%, respectively, for patients in whom TTPVI was absent (log-rank test, p < 0.001; **Figure 3A**). The 1-, 3-, and 5-year OS rates were 88.50%, 66.39%, and 57.32%, respectively, for patients in whom TTPVI was present and 95.92%, 89.57%, and 83.05%, respectively, for those in whom TTPVI was absent (log-rank test, p < 0.001; **Figure 3B**). TTPVI was significantly associated with MVI (p < 0.001) (**Supplementary Table 3**).

**Table 2:** Univariate and multivariate Cox analysis of DFS.

Variable	Univariate		Multivariate	
	HR (95% CI)	p	HR (95% CI)	p
**Age, year (>55 *vs.* ≤55)**	0.965 (0.700–1.330)	0.827	–	–
**Gender (female *vs.* male)**	1.767 (1.001–3.116)	0.050	–	–
**Cirrhosis (present *vs.* absent)**	0.987 (0.717–1.359)	0.934	–	–
**PLT, 10^9^/L (>200 *vs.* ≤200)**	0.666 (0.481–0.922)	0.014	–	–
**ALB, g/L (≤35 *vs.* >35)**	1.406 (0.838–2.359)	0.197	–	–
**ALP, U/L (>160 *vs.* ≤160)**	1.050 (0.568–1.939)	0.876	–	–
**ALT, U/L (>40 *vs.* ≤40)**	1.668 (1.217–2.288)	0.001	1.620 (1.180–2.225)	0.003
**AST, U/L (>40 *vs.* ≤40)**	1.419 (1.038–1.941)	0.028	–	–
**GGT, U/L (>75 *vs.* ≤75)**	1.519 (1.104–2.090)	0.010	–	–
**TB, μmol/L (>17.1 *vs.* ≤17.1)**	1.146 (0.820–1.603)	0.426	–	–
**PT, s (>13 *vs.* ≤13)**	1.059 (0.708–1.585)	0.779	–	–
**AFP, ng/ml (>200 *vs.* ≤200)**	1.441 (1.053–1.972)	0.022	–	–
**CA19-9, kU/L (>34 *vs.* ≤34)**	1.310 (0.925–1.856)	0.129	–	–
**Diameter, cm**	1.132 (1.080–1.187)	<0.001	–	–
**Necrosis (present *vs.* absent)**	2.193 (1.568–3.069)	<0.001	–	–
**Capsule (present *vs.* absent)**	0.413 (0.286–0.598)	<0.001	0.504 (0.341–0.745)	<0.001
**Margin (non-smooth *vs.* smooth)**	1.636 (1.196–2.236)	<0.001	–	–
**TTPVI (present *vs.* absent)**	2.368 (1.728–3.245)	<0.001	1.842 (1.319–2.572)	<0.001
**BCLC (Stage 0 *vs.* Stage A)**	1.954 (0.915–4.172)	0.083	–	–

AFP, alpha-fetoprotein; AJCC, American Joint Committee on Cancer; ALB, albumin; ALP, alkaline phosphatase; ALT, alanine aminotransferase; AST, aspartate aminotransferase; BCLC, Barcelona Clinic Liver Cancer; CA19-9, carbohydrate antigen 19-9; DFS, disease-free survival; GGT, gamma-glutamyl transpeptidase; HR, hazard ratio; PLT, platelets; PT, prothrombin time; TB, total bilirubin; TTPVI, two-trait predictor of venous invasion.

**Table 3:** Univariate and multivariate Cox analysis of OS.

Variable	Univariate		Multivariate	
	HR (95% CI)	p	HR (95% CI)	p
**Age, year (>55 *vs.* ≤55)**	0.710 (0.438–1.152)	0.165	–	–
**Gender (female *vs.* male)**	1.634 (0.710–3.761)	0.249	–	–
**Cirrhosis (present *vs.* absent)**	0.844 (0.536–1.329)	0.465	–	–
**PLT, 10^9^/L (>200 *vs.* ≤200)**	0.567 (0.359–0.897)	0.015	–	–
**ALB, g/L (≤35 *vs.* >35)**	1.898 (1.001–3.599)	0.007	–	–
**ALP, U/L (>160 *vs.* ≤160)**	1.324 (0.575–3.049)	0.510	–	–
**ALT, U/L (>40 *vs.* ≤40)**	1.703 (1.075–2.699)	0.023	–	–
**AST, U/L (>40 *vs.* ≤40)**	1.798 (1.143–2.826)	0.011	–	–
**GGT, U/L (>75 *vs.* ≤75)**	1.927 (1.227–3.026)	0.004	1.787 (1.134–2.814)	0.012
**TB, μmol/L (>17.1 *vs.* ≤17.1)**	1.030 (0.6275–1.692)	0.907	–	–
**PT, s (>13 *vs.* ≤13)**	1.015 (0.558–1.843)	0.962	–	–
**AFP, ng/ml (>200 *vs.* ≤200)**	2.273 (1.444–3.580)	<0.001	1.722 (1.067–2.788)	0.026
**CA19-9, kU/L (>34 *vs.* ≤34)**	1.448 (0.892–2.348)	0.134	–	–
**Diameter, cm**	1.159 (1.084–1.240)	<0.001	–	–
**Necrosis (present *vs.* absent)**	2.737 (1.627–4.605)	<0.001	–	–
**Capsule (present *vs.* absent)**	0.420 (0.242–0.729)	<0.001	–	–
**Margin (non-smooth *vs.* smooth)**	2.143 (1.360–3.376)	<0.001	–	–
**TTPVI (present *vs.* absent)**	3.133 (1.948–5.038)	<0.001	2.509 (1.518–4.147)	<0.001
**BCLC (Stage 0 *vs.* Stage A)**	2.900 (0.712–11.816)	0.137	–	–

AFP, alpha-fetoprotein; AJCC, American Joint Committee on Cancer; ALB, albumin; ALP, alkaline phosphatase; ALT, alanine aminotransferase; AST, aspartate aminotransferase; BCLC, Barcelona Clinic Liver Cancer; CA19-9, carbohydrate antigen 19-9; GGT, gamma-glutamyl transpeptidase; HR, hazard ratio; OS, overall survival; PLT, platelets; PT, prothrombin time; TB, total bilirubin; TTPVI, two-trait predictor of venous invasion.

### Subgroup Analyses

**Figure 4:**
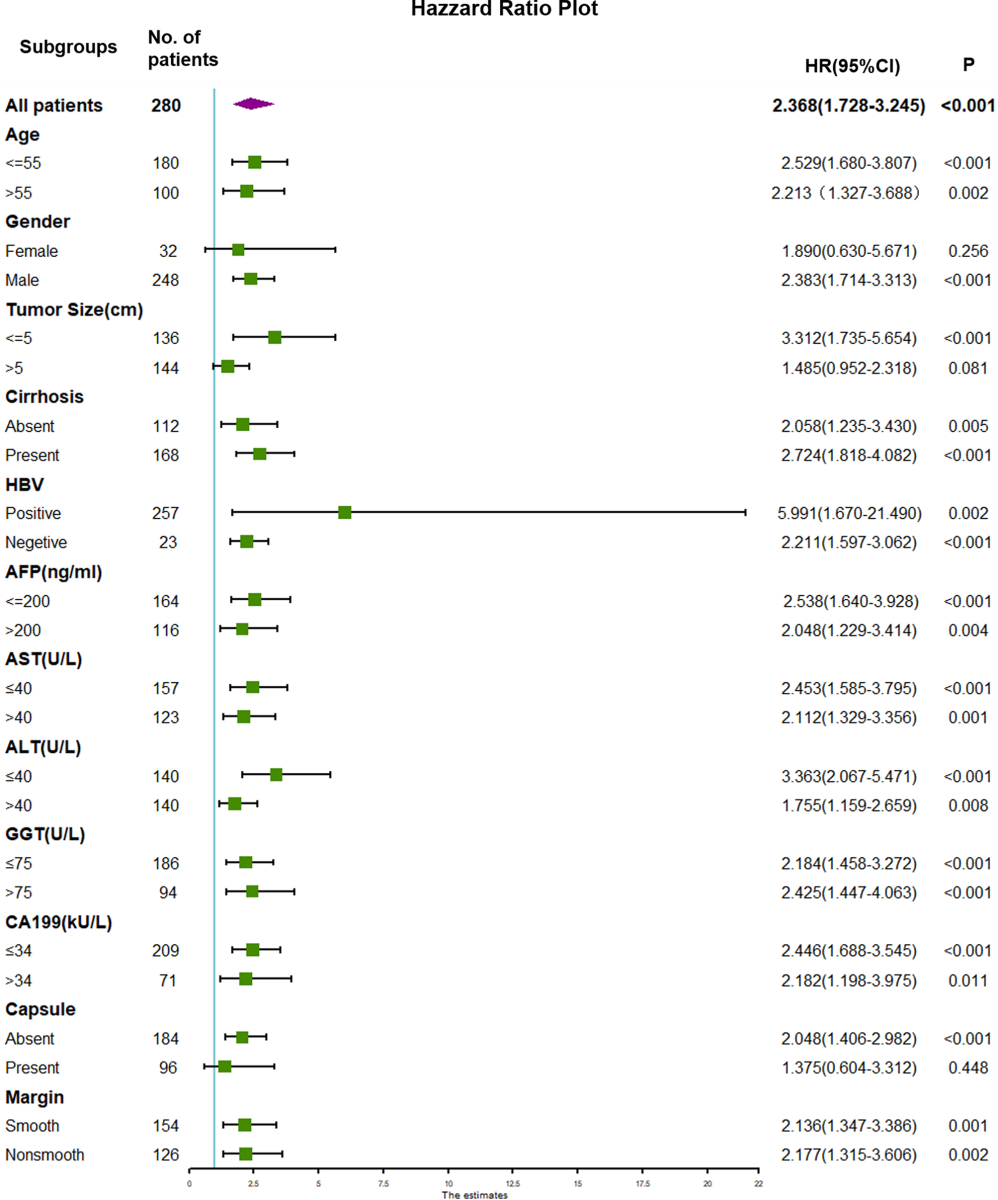
Forest plot of hazard ratios (HRs) for TTPVI present versus TTPVI absent of hepatocellular carcinoma in the subgroup analysis of disease-free survival. TTPVI, two-trait predictor of venous invasion.

**Figure 5:**
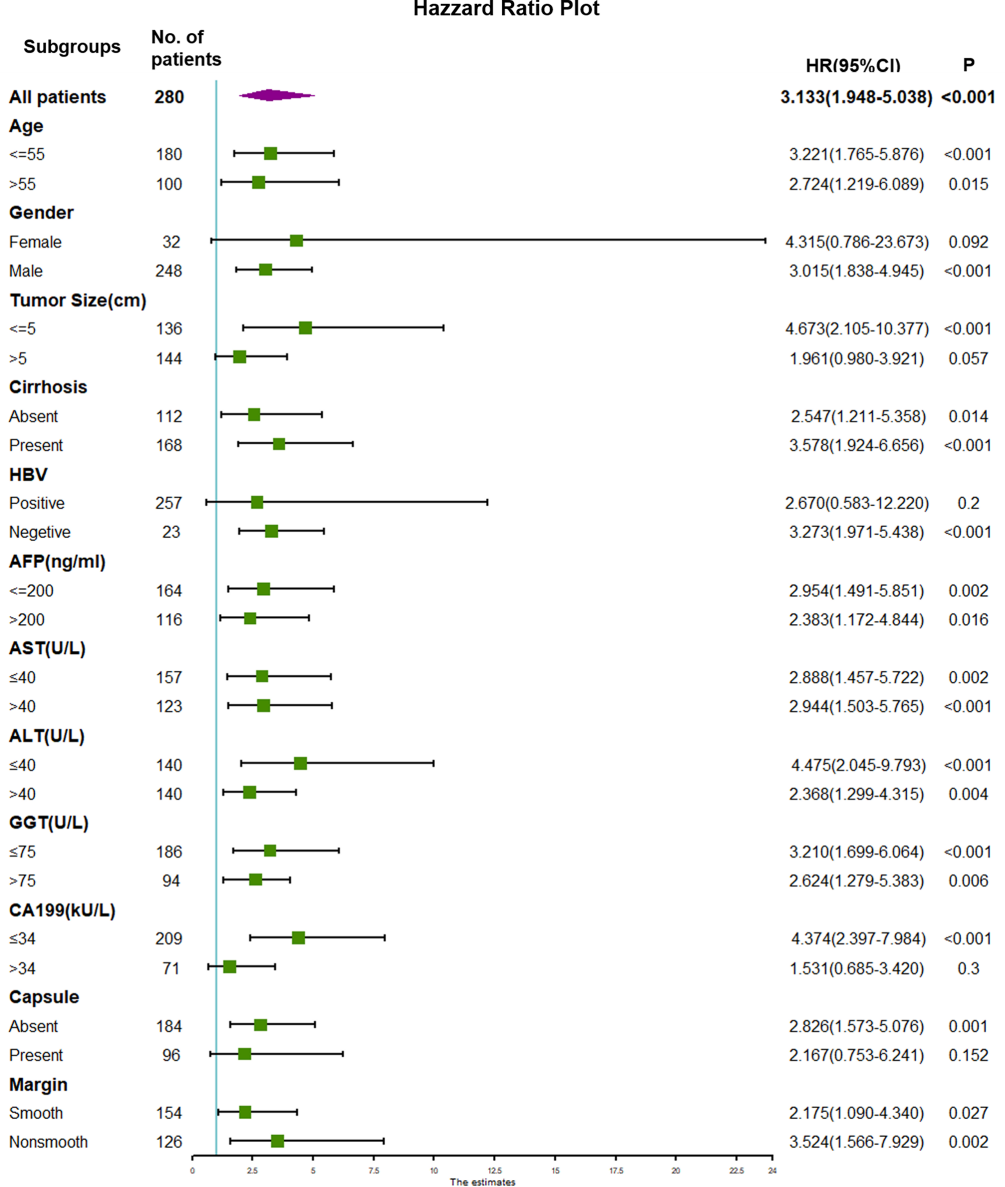
Forest plot of hazard ratios (HRs) for TTPVI present versus TTPVI absent of hepatocellular carcinoma in the subgroup analysis of overall survival. TTPVI, two-trait predictor of venous invasion.

Subgroup analyses were performed to further investigate the preoperative significance of TTPVI. Forest plots displaying the impact of TTPVI are shown in **Figures 4** and **5**. There was no interaction detected between TTPVI and these factors. However, the presence of TTPVI indicated a lower rate of DFS and OS in most subgroups.

**Figure 6:**
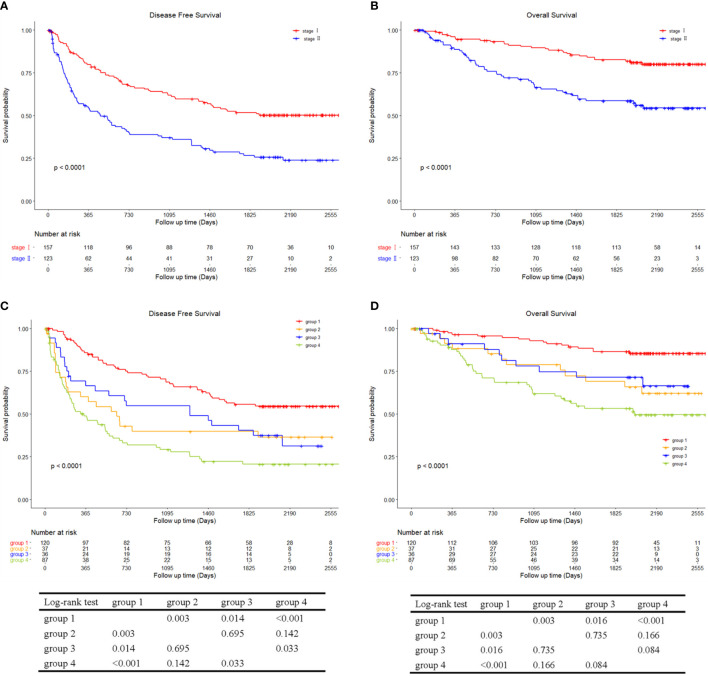
Kaplan–Meier curves of disease-free survival and overall survival between AJCC stage I and stage II and stratified into four groups using a combination of TTPVI on preoperative contrast-enhanced computed tomography. **(A)** Disease-free survival and **(B)** overall survival curves between AJCC stage I and stage II. **(C)** Disease-free survival and **(D)** overall survival curves comparing surgical resection stratified into four groups using a combination of independent factors (AJCC stage and TTPVI). AJCC, American Joint Committee on Cancer; TTPVI, two-trait predictor of venous invasion.

**Figure 7:**
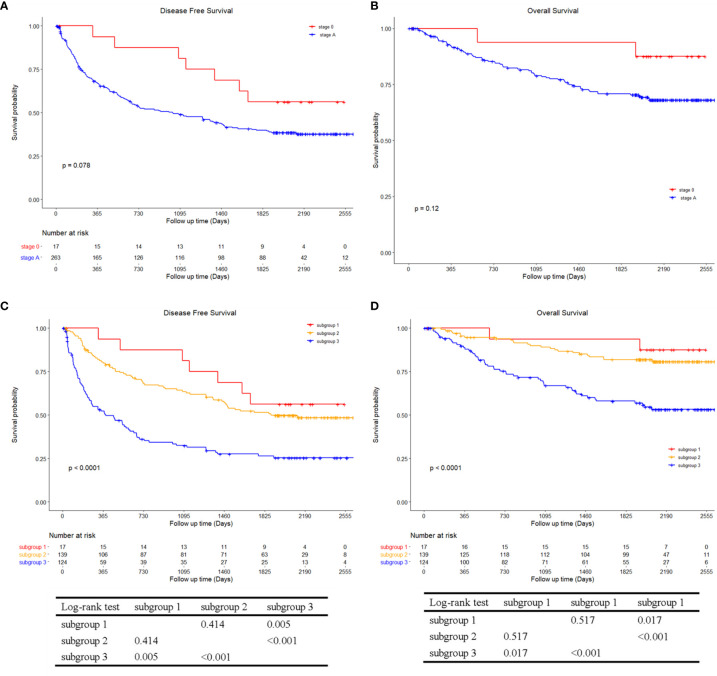
Kaplan–Meier curves of disease-free survival and overall survival between BCLC stage 0 and stage A stratified into three groups using a combination of TTPVI on preoperative contrast-enhanced computed tomography. **(A)** Disease-free survival and **(B)** overall survival curves between BCLC stage 0 and stage **(A, C)** Disease-free survival and **(D)** overall survival curves comparing surgical resection recipients stratified into three groups using a combination of independent factors (BCLC stage and TTPVI). There were no patients with TTPVI in BCLC stage 0. BCLC, Barcelona Clinic Liver Cancer; TTPVI, two-trait predictor of venous invasion.

DFS and OS according to the AJCC and BCLC stage and the corresponding stratified analysis are shown in **Figures 6**, **7**, respectively. Patients in stage II had a lower DFS (p < 0.001; **Figure 6A**) and OS (p < 0.001; **Figure 6B**) than patients in stage I. There were no significant differences in DFS (p = 0.078; **Figure 7A**) and OS (p = 0.120; **Figure 7B**) between patients in stage BCLC 0 and BCLC A. **Figure 6** shows the comparison in DFS and OS between patients with TTPVI absent in stage I (group 1, 120 patients) and stage II (group 3, 36 patients) and patients with TTPVI present in stage I (group 2, 37 patients) and stage II (group 4, 87 patients). Interestingly, patients in groups 2 and 4 had lower DFS (**Figure 6C**) and OS (**Figure 6D**) rates than those in groups 1 and 3. Moreover, patients in whom TTPVI was present in stage I had a similar DFS (p = 0.695, **Figure 6C**) and OS (p = 0.735, **Figure 6D**) compared with patients in whom TTPVI was absent in stage II. **Figure 5** shows the comparison in DFS and OS between patients in whom TTPVI was absent in BCLC 0 (subgroup 1, 17 patients), BCLC A (subgroup 2, 139 patients), and patients in whom TTPVI was present in BCLC A (subgroup 3, 124 patients). There were significant differences in DFS (p < 0.001; **Figure 7C**) and OS (p < 0.001; **Figure 7D**) between subgroups 2 and 3. There were no patients with TTPVI in BCLC stage 0.

## Discussion

In the present study, we sought to investigate the ability of TTPVI detected on preoperative CECT to predict DFS and OS after surgical resection in patients with early-stage HCC. We found that TTPVI is an independent preoperative predictor of early-stage HCC after hepatectomy. Furthermore, it remained a strong independent preoperative predictor of survival even after adjusting for other clinical and imaging variables. Moreover, TTPVI can provide added prognostic information when integrated into the BCLC and AJCC staging systems.

Identification of poorly differentiated HCC plays an important role in ensuring effective therapeutic progress and improving the management of these patients. In addition to its roles in diagnosis and localization, CECT may also be used to characterize tumor biology. In the present study, the univariate analysis indicated that diameter, necrosis, and margin were associated with DFS and OS, which is consistent with several previous reports (20–24).

In our study, the multivariate analysis indicated that TTPVI was an independent risk factor of poor DFS and OS. Moreover, the combined use of the BCLC and AJCC staging system (morphological information) and TTPVI on preoperative CECT (angiogenesis and cellular proliferation information) improved the stratification of patients in terms of the risk of a poor prognosis after hepatectomy. To improve prognosis in patients undergoing surgical resection for HCC, postoperative adjuvant therapies are considered to be effective strategies (25). Therefore, patients with TTPVI in AJCC stage II or BCLC A may require a more meticulous follow-up plan for early identification of the recurrence of HCCs. Importantly, TTPVI is associated with a specific HCC molecular profile, derived from a microscopic venous invasion gene profile correlated with angiogenesis, cell proliferation, and MVI (11, 12). To the best of our knowledge, this is the first study reporting TTPVI as an independent preoperative predictor of DFS and OS in patients with early-stage HCC. However, radiogenomic venous invasion, which is a little different from TTPVI, defined as when internal arteries and tumor-liver differences are observed in the absence of a hypodense halo, has been reported as a significant predictor of poor prognosis in several previous studies (20, 22, 26). This further indicates that it is reasonable to assume that TTPVI is an important preoperative factor for survival.

In our study, HCC with a capsule appearance was a favorable prognostic factor in both the univariate and multivariate analyses, as the DFS rates in these patients were higher than those without capsule appearance. This is in accordance with several previous reports (24, 27, 28). We also found that HCC with a capsule appearance was related to lower recurrence rates after surgery or ablation and more effective transcatheter embolization. This may be partly due to the barrier impact of the fibrous capsule that hinders HCC invasion. In addition, an intact capsule appearance observed by CT is indicative of a lower probability of MVI being present in the surrounding liver parenchyma (29, 30).

In this study, the multivariate analysis of AFP was adjusted for clinical variables for OS confirmed in previous reports (31, 32). Notably, preoperative serum AFP may be an indirect indicator of tumor burden. In fact, several studies have reported that an elevated AFP serum level is one of the crucial factors heralding poor survival after surgical resection, radiofrequency ablation, and trans-catheter arterial chemoembolization of HCC (31–34).

In this study, both the univariate and multivariate analyses revealed that an elevated level of GGT was a prognosis predictor for patients with HCC after curative resection over a long-term follow-up, and GGT was significantly correlated with shorter OS, which is consistent with several previous reports (35–37). In China, most HCCs develop in parallel to an underlying HBV infection, which results in the accumulation of chronic liver injury. In our study, which included 280 patients with HCC, 257 patients were hepatitis B surface antigen positive. Furthermore, it has been demonstrated that the occurrence and progression of HCC are closely associated with inflammatory and immune factors. Previous studies have indicated that high expression levels of GGT could break the oxidant/antioxidant balance, subsequently leading to persistent oxidative stress in the tumor thus promoting tumor progression (38). Furthermore, as a marker of the inflamed liver microenvironment in patients with hepatitis, GGT plays a critical role in tumor progression and metastasis (39, 40). These previously identified mechanisms may support our finding that GGT has predictive value for patients with early-stage HCC following hepatectomy.

This study had several limitations. First, its retrospective design is a potential source of selection bias. Therefore, more prospective studies including larger numbers of patients are needed to validate our results. Second, our cohort largely included patients with HBV; therefore, our results may not be broadly applicable to patients with other liver diseases. Third, in the subgroup analysis, there were no patients with TTPVI in BCLC stage 0, and the population with an absence of TTPVI in BCLC stage 0 was small; thus, future studies to include more patients in BCLC stage 0 are needed. Four, a clinical-radiomic analysis have not been performed for prediction prognosis. Finally, the prognostic value of TTPVI was not assessed in patients undergoing other therapies, such as radiofrequency ablation and trans-catheter arterial chemoembolization.

In conclusion, TTPVI detected with CECT can serve as a preoperative prognostic marker for early-stage HCC after hepatectomy. Moreover, CECT images obtained at the time of diagnosis of HCC can provide additional information on the prognosis of patients with early-stage HCC.

## Data Availability Statement

The raw data supporting the conclusions of this article will be made available by the authors, without undue reservation.

## Ethics Statement

This retrospective clinical study was approved by our institutional ethics review board of Zhujiang Hospital of Southern Medical University (2019-KY-021-01) and Sun Yat-sen University Cancer Center (B2021-214-01), and the requirement for written informed consent was waived. Written informed consent for participation was not required for this study in accordance with the national legislation and the institutional requirements.

## Author Contributions

XL, WL, ZQ, ZG, and XQ: conception, design, statistical analysis and manuscript writing. XZ, ZL, SQ, MY, QK, XJ, TL, and MZ: data collection and data interpretation. CX and XQ resource and study supervision. All authors contributed to the article and approved the submitted version.

## Funding

This work was funded by Guangdong Basic and Applied Basic Research Foundation (2019A1515011269 and 2021A1515011305) and Clinical Research Starup Program of Southern Medical University by High-Level University Construction Funding of Guangdong Provincial Department of Education (LC2016PY034).

## Conflict of Interest

The authors declare that the research was conducted in the absence of any commercial or financial relationships that could be construed as a potential conflict of interest.

## Publisher’s Note

All claims expressed in this article are solely those of the authors and do not necessarily represent those of their affiliated organizations, or those of the publisher, the editors and the reviewers. Any product that may be evaluated in this article, or claim that may be made by its manufacturer, is not guaranteed or endorsed by the publisher.

## Abbreviations

AFP, alpha-fetoprotein; AJCC, American Joint Committee on Cancer; BCLC, Barcelona Clinic Liver Cancer; CECT, contrast-enhanced computed tomography; CI, confidence interval; DFS, disease-free survival; GGT, gamma-glutamyl transpeptidase; HBV, hepatitis B virus; HCC, hepatocellular carcinoma; HR, hazard ratios; MVI, microvascular invasion; OS, overall survival; TTPVI, two-trait predictor of venous invasion.

## References

[1] Bray F Ferlay J Soerjomataram I Siegel RL Torre LA Jemal A. Global Cancer Statistics 2018: GLOBOCAN Estimates of Incidence and Mortality Worldwide for 36 Cancers in 185 Countries. CA Cancer J Clin (2018) 68:394–424. 10.3322/caac.21492 30207593

[2] Tsilimigras DI Bagante F Sahara K Moris D Hyer JM Wu L. Prognosis After Resection of Barcelona Clinic Liver Cancer (BCLC) Stage 0, A, and B Hepatocellular Carcinoma: A Comprehensive Assessment of the Current BCLC Classification. Ann Surg Oncol (2019) 26:3693–700. 10.1245/s10434-019-07580-9 31267302

[3] Cai W He B Hu M Zhang W Xiao D Yu H. A Radiomics-Based Nomogram for the Preoperative Prediction of Posthepatectomy Liver Failure in Patients With Hepatocellular Carcinoma. Surg Oncol (2019) 28:78–85. 10.1016/j.suronc.2018.11.013 30851917

[4] Bruix J Gores GJ Mazzaferro V. Hepatocellular Carcinoma: Clinical Frontiers and Perspectives. Gut (2014) 63:844–55. 10.1136/gutjnl-2013-306627 PMC4337888 24531850

[5] Park S Choi S Cho YA Sinn DH Kim JM Park CK. Evaluation of the American Joint Committee on Cancer (AJCC) 8th Edition Staging System for Hepatocellular Carcinoma in 1,008 Patients With Curative Resection. Cancer Res Treat (2020) 52:1145–52. 10.4143/crt.2020.208 PMC7577811 32599989

[6] Li X Qi Z Du H Geng Z Li Z Qin S. Deep Convolutional Neural Network for Preoperative Prediction of Microvascular Invasion and Clinical Outcomes in Patients with HCCs. Eur Radiol (2021). 10.1007/s00330-021-08198-w 34347160

[7] Li H Zhang J Zheng Z Guo Y Chen M Xie C. Preoperative Histogram Analysis of Intravoxel Incoherent Motion (IVIM) for Predicting Microvascular Invasion in Patients With Single Hepatocellular Carcinoma. Eur J Radiol (2018) 105:65–71. 10.1016/j.ejrad.2018.05.032 30017300

[8] European Association for the Study of the Liver. Electronic Address Eee, European Association for the Study of the L. EASL Clinical Practice Guidelines: Management of Hepatocellular Carcinoma. J Hepatol (2018) 69:182–236. 10.1016/j.jhep.2018.03.019 29628281

[9] Xie DY Ren ZG Zhou J Fan J Gao Q. 2019 Chinese Clinical Guidelines for the Management of Hepatocellular Carcinoma: Updates and Insights. Hepatobiliary Surg Nutr (2020) 9:452–63. 10.21037/hbsn-20-480 PMC7423548 32832496

[10] Li M Xin Y Fu S Liu Z Li Y Hu B. Corona Enhancement and Mosaic Architecture for Prognosis and Selection Between of Liver Resection Versus Transcatheter Arterial Chemoembolization in Single Hepatocellular Carcinomas >5 cm Without Extrahepatic Metastases: An Imaging-Based Retrospective Study. Med (Baltimore) (2016) 95:e2458. 10.1097/MD.0000000000002458 PMC4718267 26765441

[11] Segal E Sirlin CB Ooi C Adler AS Gollub J Chen X. Decoding Global Gene Expression Programs in Liver Cancer by Noninvasive Imaging. Nat Biotechnol (2007) 25:675–80. 10.1038/nbt1306 17515910

[12] Renzulli M Brocchi S Cucchetti A Mazzotti F Mosconi C Sportoletti C. Can Current Preoperative Imaging Be Used to Detect Microvascular Invasion of Hepatocellular Carcinoma? Radiology (2016) 279:432–42. 10.1148/radiol.2015150998 26653683

[13] Peng J Zhang J Zhang Q Xu Y Zhou J Liu L. A Radiomics Nomogram for Preoperative Prediction of Microvascular Invasion Risk in Hepatitis B Virus-Related Hepatocellular Carcinoma. Diagn Interv Radiol (2018) 24:121–7. 10.5152/dir.2018.17467 PMC5951199 29770763

[14] Zhang T Pandey G Xu L Chen W Gu L Wu Y. The Value of TTPVI in Prediction of Microvascular Invasion in Hepatocellular Carcinoma. Cancer Manag Res (2020) 12:4097–105. 10.2147/CMAR.S245475 PMC7276193 32581583

[15] Bakr S Gevaert O Patel B Kesselman A Shah R Napel S. Interreader Variability in Semantic Annotation of Microvascular Invasion in Hepatocellular Carcinoma on Contrast-Enhanced Triphasic CT Images. Radiol Imaging Cancer (2020) 2:e190062. 10.1148/rycan.2020190062 32550600 PMC7263284

[16] Sun SW Liu QP Xu X Zhu FP Zhang YD Liu XS. Direct Comparison of Four Presurgical Stratifying Schemes for Prediction of Microvascular Invasion in Hepatocellular Carcinoma by Gadoxetic Acid-Enhanced MRI. J Magn Reson Imaging (2020) 52:433–47. 10.1002/jmri.27043 31943465

[17] Forner A Reig ME de Lope CR Bruix J. Current Strategy for Staging and Treatment: The BCLC Update and Future Prospects. Semin Liver Dis (2010) 30:61–74. 10.1055/s-0030-1247133 20175034

[18] Finn RS Zhu AX Farah W Almasri J Zaiem F Prokop LJ. Therapies for Advanced Stage Hepatocellular Carcinoma With Macrovascular Invasion or Metastatic Disease: A Systematic Review and Meta-Analysis. Hepatology (2018) 67:422–35. 10.1002/hep.29486 28881497

[19] Lee S Kim SH Lee JE Sinn DH Park CK. Preoperative Gadoxetic Acid-Enhanced MRI for Predicting Microvascular Invasion in Patients With Single Hepatocellular Carcinoma. J Hepatol (2017) 67:526–34. 10.1016/j.jhep.2017.04.024 28483680

[20] Banerjee S Wang DS Kim HJ Sirlin CB Chan MG Korn RL. A Computed Tomography Radiogenomic Biomarker Predicts Microvascular Invasion and Clinical Outcomes in Hepatocellular Carcinoma. Hepatology (2015) 62:792–800. 10.1002/hep.27877 25930992 PMC4654334

[21] Brenet Defour L Mule S Tenenhaus A Piardi T Sommacale D Hoeffel C. Hepatocellular Carcinoma: CT Texture Analysis as a Predictor of Survival After Surgical Resection. Eur Radiol (2019) 29:1231–9. 10.1007/s00330-018-5679-5 30159621

[22] Ji GW Zhu FP Xu Q Wang K Wu MY Tang WW. Machine-Learning Analysis of Contrast-Enhanced CT Radiomics Predicts Recurrence of Hepatocellular Carcinoma After Resection: A Multi-Institutional Study. EBioMedicine (2019) 50:156–65. 10.1016/j.ebiom.2019.10.057 PMC6923482 31735556

[23] Shim JH Jun MJ Han S Lee YJ Lee SG Kim KM. Prognostic Nomograms for Prediction of Recurrence and Survival After Curative Liver Resection for Hepatocellular Carcinoma. Ann Surg (2015) 261:939–46. 10.1097/SLA.0000000000000747 24950276

[24] Fu YP Yi Y Huang JL Jing CY Sun J Ni XC. Prognostic Nomograms Stratify Survival of Patients With Hepatocellular Carcinoma Without Portal Vein Tumor Thrombosis After Curative Resection. Oncologist (2017) 22:561–9. 10.1634/theoncologist.2016-0231 PMC5423510 28438885

[25] Wang YY Wang LJ Xu D Liu M Wang HW Wang K. Postoperative Adjuvant Transcatheter Arterial Chemoembolization Should be Considered Selectively in Patients Who Have Hepatocellular Carcinoma With Microvascular Invasion. HPB (Oxf) (2019) 21:425–33. 10.1016/j.hpb.2018.08.001 30249510

[26] Zhang W Chen J Liu L Wang L Liu J Su D. Prognostic Value of Preoperative Computed Tomography in HBV-Related Hepatocellular Carcinoma Patients After Curative Resection. Onco Targets Ther (2019) 12:3791–804. 10.2147/OTT.S199136 PMC6529036 31190879

[27] Lim JH Jang HJ Kim EY Park CK Joh JW Kim YI. Early Recurring Hepatocellular Carcinoma After Partial Hepatic Resection: Preoperative CT Findings. Korean J Radiol (2000) 1:38–42. 10.3348/kjr.2000.1.1.38 11752927 PMC2718136

[28] Lai EC Ng IO Ng MM Lok AS Tam PC Fan ST. Long-Term Results of Resection for Large Hepatocellular Carcinoma: A Multivariate Analysis of Clinicopathological Features. Hepatology (1990) 11:815–8. 10.1002/hep.1840110516 2161393

[29] Cho ES Choi JY. MRI Features of Hepatocellular Carcinoma Related to Biologic Behavior. Korean J Radiol (2015) 16:449–64. 10.3348/kjr.2015.16.3.449 PMC4435980 25995679

[30] Lim JH Choi D Park CK Lee WJ Lim HK. Encapsulated Hepatocellular Carcinoma: CT-Pathologic Correlations. Eur Radiol (2006) 16:2326–33. 10.1007/s00330-006-0203-8 16547706

[31] Zhuang H Zhou Z Ma Z Huang S Gong Y Zhang Z. Prognostic Stratification Based on a Novel Nomogram for Solitary Large Hepatocellular Carcinoma After Curative Resection. Front Oncol (2020) 10:556489. 10.3389/fonc.2020.556489 33312945 PMC7703492

[32] Chan MY She WH Dai WC Tsang SHY Chok KSH Chan ACY. Prognostic Value of Preoperative Alpha-Fetoprotein (AFP) Level in Patients Receiving Curative Hepatectomy- An Analysis of 1,182 Patients in Hong Kong. Transl Gastroenterol Hepatol (2019) 4:52. 10.21037/tgh.2019.06.07 31463411 PMC6691082

[33] Choi D Lim HK Rhim H Kim YS Lee WJ Paik SW. Percutaneous Radiofrequency Ablation for Early-Stage Hepatocellular Carcinoma as a First-Line Treatment: Long-Term Results and Prognostic Factors in a Large Single-Institution Series. Eur Radiol (2007) 17:684–92. 10.1007/s00330-006-0461-5 17093964

[34] Chen M Cao J Hu J Topatana W Li S Juengpanich S. Clinical-Radiomic Analysis for Pretreatment Prediction of Objective Response to First Transarterial Chemoembolization in Hepatocellular Carcinoma. Liver Cancer (2021) 10:38–51. 10.1159/000512028 33708638 PMC7923935

[35] Liao M Qin W Liao Y Yao R Yu J Liao W. Prognostic Value of Gamma-Glutamyl Transpeptidase to Lymphocyte Count Ratio in Patients With Single Tumor Size </= 5 cm Hepatocellular Carcinoma After Radical Resection. Front Oncol (2019) 9:347. 10.3389/fonc.2019.00347 31165038 PMC6536585

[36] Zhang CH Ni XC Chen BY Qiu SJ Zhu YM Luo M. Combined Preoperative Albumin-Bilirubin (ALBI) and Serum Gamma-Glutamyl Transpeptidase (GGT) Predicts the Outcome of Hepatocellular Carcinoma Patients Following Hepatic Resection. J Cancer (2019) 10:4836–45. 10.7150/jca.33877 PMC6775507 31598154

[37] Wu SJ Lin YX Ye H Xiong XZ Li FY Cheng NS. Prognostic Value of Alkaline Phosphatase, Gamma-Glutamyl Transpeptidase and Lactate Dehydrogenase in Hepatocellular Carcinoma Patients Treated With Liver Resection. Int J Surg (Lond Engl) (2016) 36:143–51. 10.1016/j.ijsu.2016.10.033 27793641

[38] Corti A Franzini M Paolicchi A Pompella A. Gamma-Glutamyltransferase of Cancer Cells at the Crossroads of Tumor Progression, Drug Resistance and Drug Targeting. Anticancer Res (2010) 30:1169–81. 10.1096/fasebj.23.1_supplement.438.6 20530424

[39] Sun P Li Y Chang L Tian X. Prognostic and Clinicopathological Significance of Gamma-Glutamyltransferase in Patients With Hepatocellular Carcinoma: A PRISMA-Compliant Meta-Analysis. Med (Baltimore) (2019) 98:e15603. 10.1097/MD.0000000000015603 PMC6531078 31083251

[40] Everhart JE Wright EC. Association of Gamma-Glutamyl Transferase (GGT) Activity With Treatment and Clinical Outcomes in Chronic Hepatitis C (HCV). Hepatology (2013) 57:1725–33. 10.1002/hep.26203 PMC3624035 23258530

