# Supplementary material for: Two-Trait Predictor of Venous Invasion on Contrast-Enhanced CT as a Preoperative Predictor of Outcomes for Early-Stage Hepatocellular Carcinoma After Hepatectomy
Source: Front Oncol. 2021 Sep 1;11:688087. doi: 10.3389/fonc.2021.688087 (PMC8442625; doi:10.3389/fonc.2021.688087)
Supplement: Supplementary file 1 [file DataSheet_1.docx]

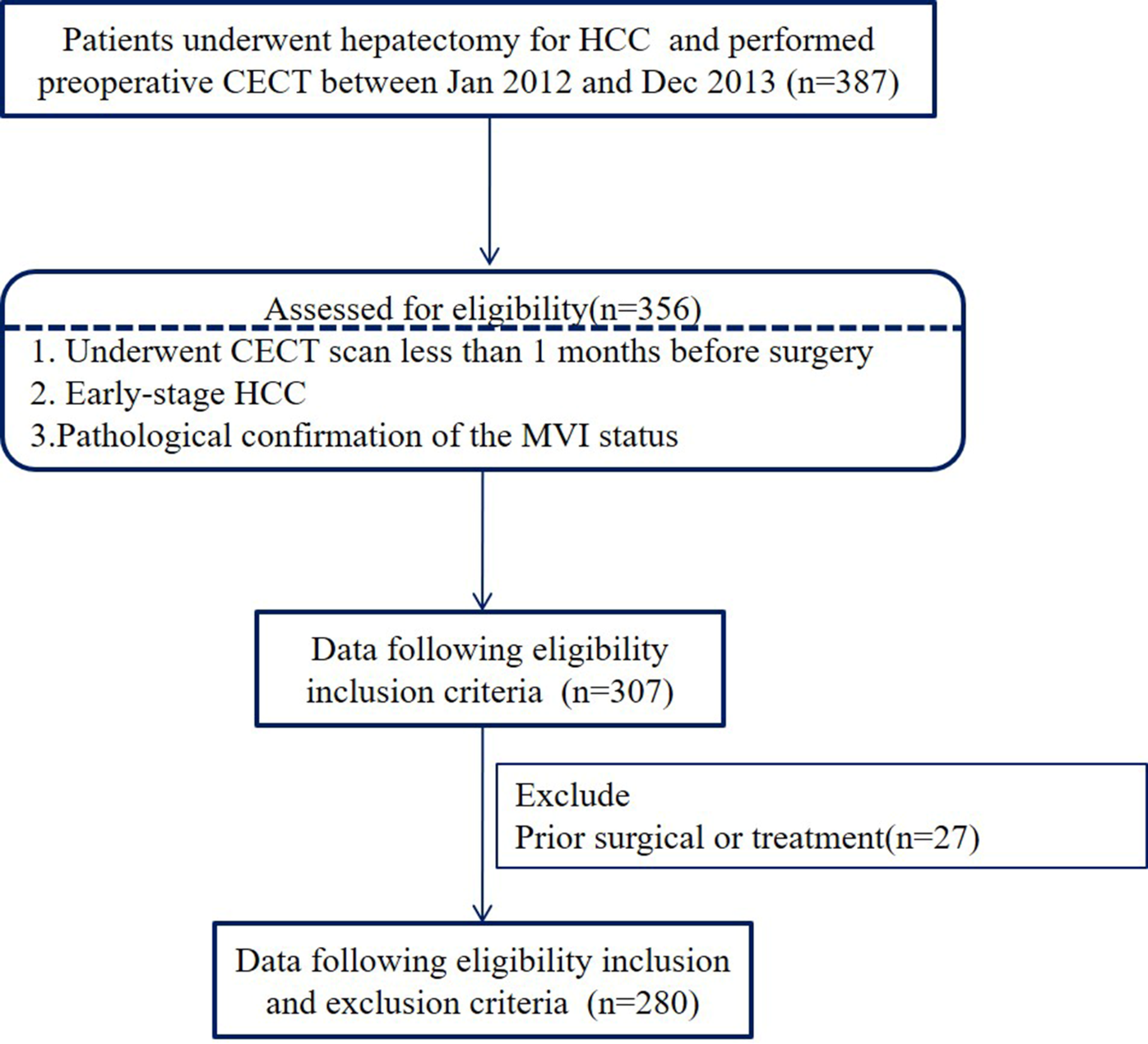


**Supplementary Figure 1.** Flow chart of this study. CECT, contrast-enhanced computed tomography; HCC, hepatocellular carcinoma; MVI, microvascular invasion.


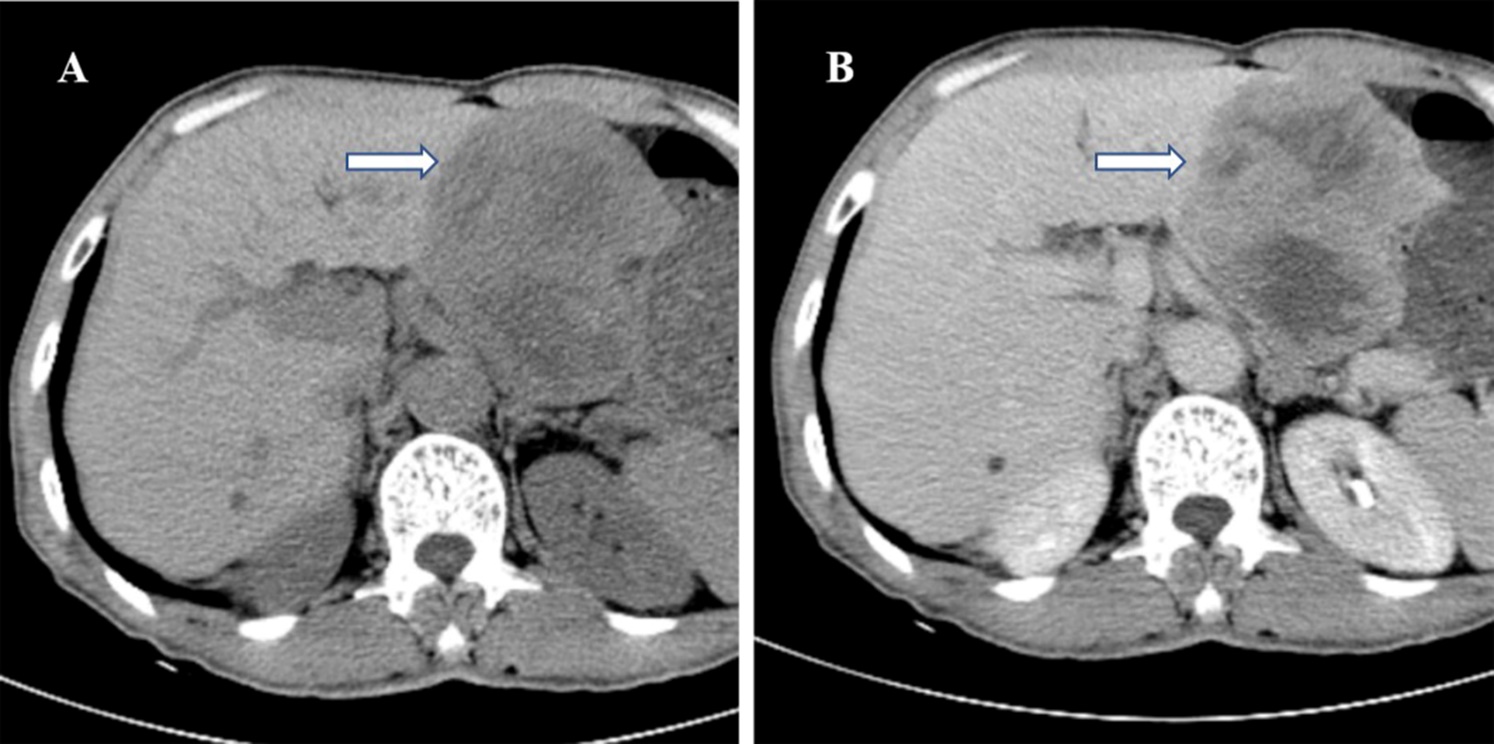


**Supplementary Figure 2.** Intratumoral necrosis.


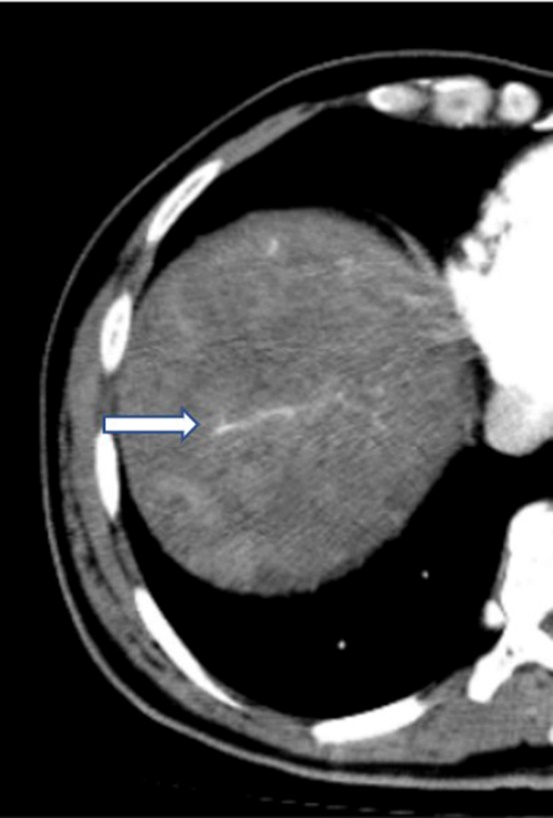


**Supplementary Figure 3.** Internal arteries.


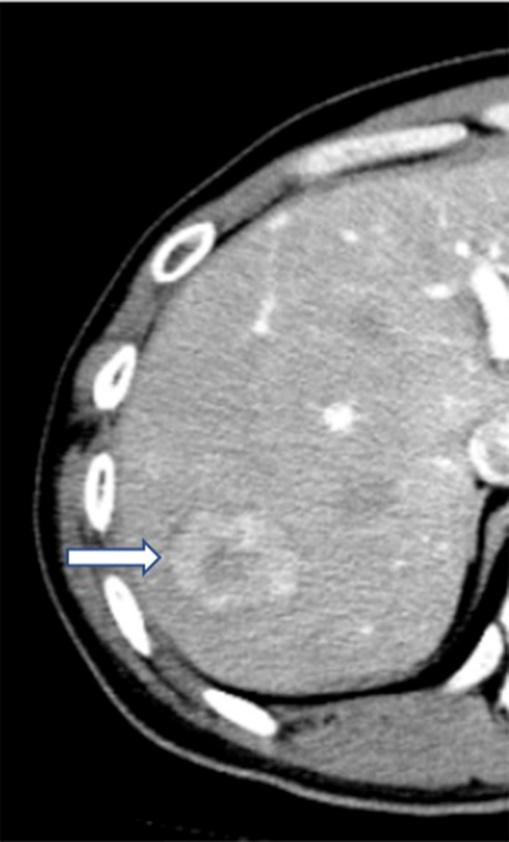


**Supplementary Figure 4.** Hypoattenuating halo, a rim of low density around the tumor.


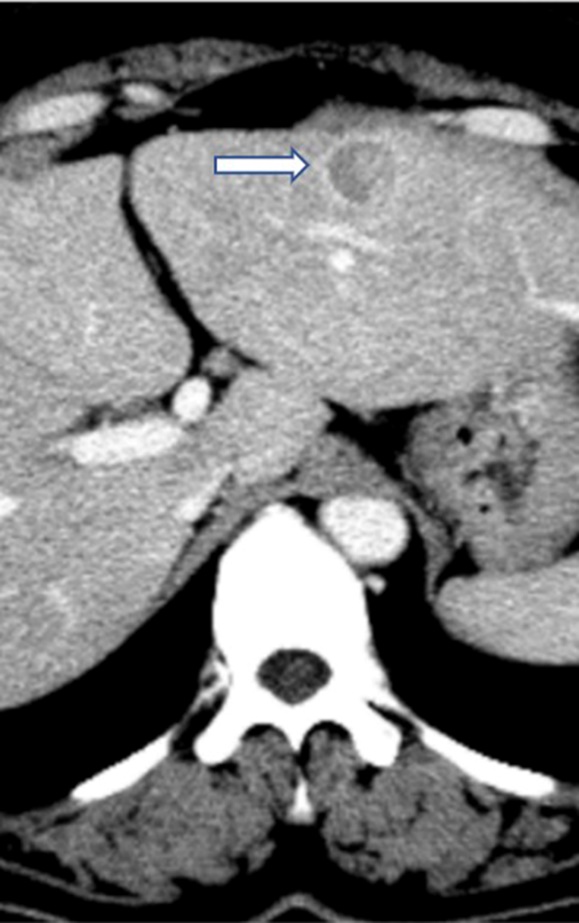


**Supplementary Figure 5.** Radiologic capsule appearance.


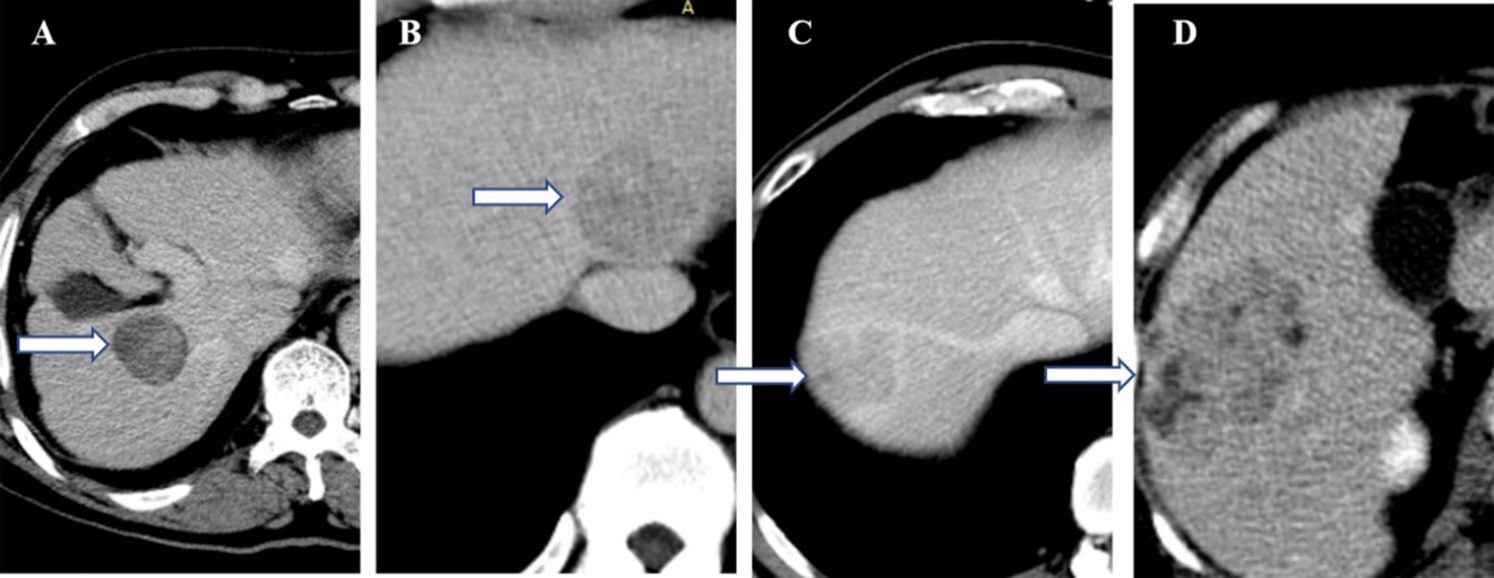


**Supplementary Figure 6.** Margin. (A) nodular-shaped tumor; (B) nodular with extranodular extension A and B were classified as smooth margin; (C) multinodular confluent; (D) infiltrative shaped tumor; C and D were classified as nonsmooth margin.


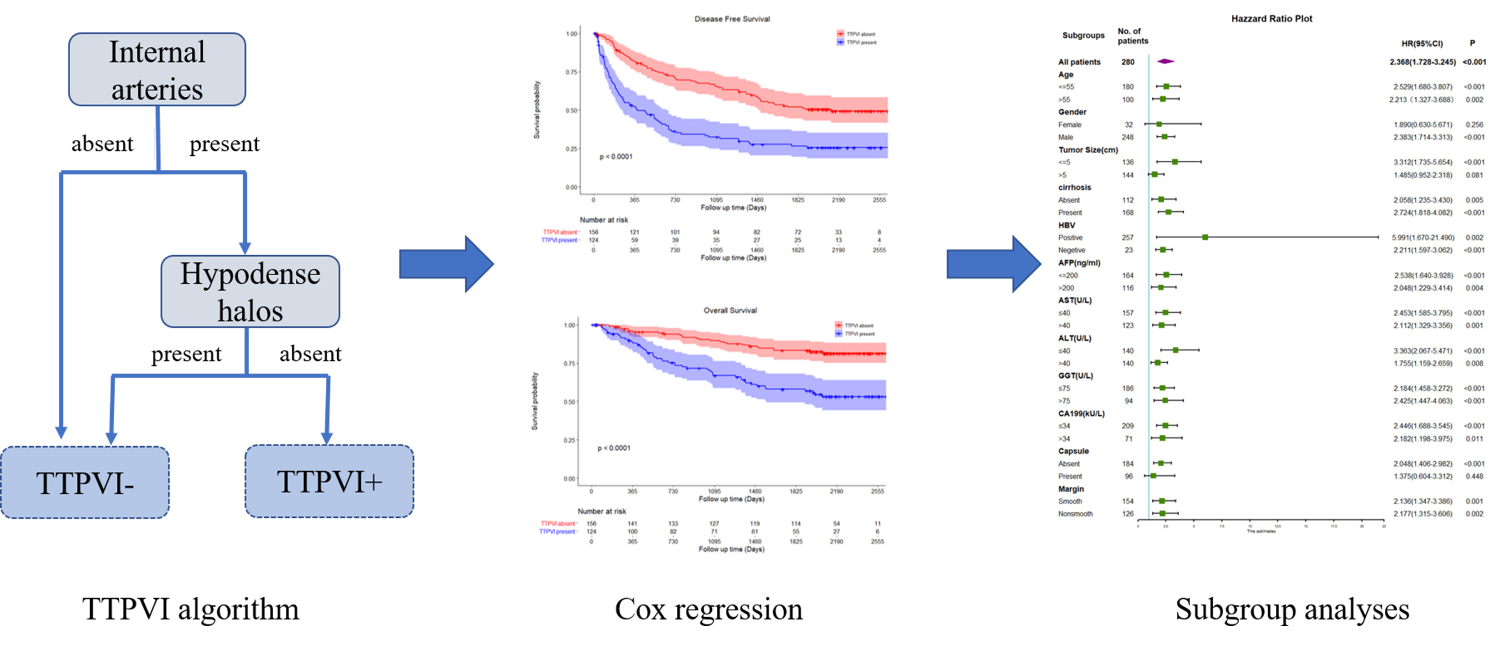


**Supplementary Figure 7.** Graphic abstract.

**Supplementary Table 1.** Imaging parameters of CECT for preoperative assessment of HCC

| **CT scanner** | **128-slice spiral CT (Aquilion TSX-101A, Toshiba)** | **256-slice spiral CT (Brilliance iCT, Philips)** | **64-slice spiral CT (Brilliance 64, Philips)** |
| --- | --- | --- | --- |
| **Tube voltage** | 120kV | 120kV | 120kV |
| **Tube current** | 250mA | 300mA | 300mA |
| **Matrix** | 512×512 | 512×512 | 512×512 |
| **Section thickness** | 5mm | 5mm | 5mm |
| **Section interval** | 5mm | 5mm | 5mm |
| **Helical pitch** | 1.0 | 0.993 | 0.984 |

CECT, contrast-enhanced computed tomography; HCC, hepatocellular carcinoma

**Supplementary Table 2.** Interobserver agreement of CT imaging features

| **Imaging features** | **Necrosis** | **Internal arteries** | **Halo** | **Capsule appearance** | **Margin** | **TTPVI** |
| --- | --- | --- | --- | --- | --- | --- |
| **Kappa**  **(95% CI)** | 0.818  (0.749-0.887) | 0.843  (0.779-0.907) | 0.748  (0.660-0.836) | 0.808  (0.737-0.879) | 0.799  (0.728-0.870) | 0.787  (0.714-0.860) |

TTPVI, two-trait predictor of venous invasion; CI, confidence index.

**Supplementary Table 3.** Relationship between TTPVI and MVI

|  | **TTPVI present (n=124)** | **TTPVI absent (n=156)** | **P value** |
| --- | --- | --- | --- |
| **MVI present** | 83 | 32 | 0.001* |
| **MVI absent** | 41 | 124 |  |

MVI, microvascular invasion, TTPVI, two-trait predictor of venous invasion.

*Statistically significant results from chi-square test.
